# Delayed Sleep in Winter Related to Natural Daylight Exposure among Arctic Day Workers

**DOI:** 10.3390/clockssleep1010010

**Published:** 2018-11-30

**Authors:** Arne Lowden, Nelson A. M. Lemos, Bruno S. B. Gonçalves, Gülçin Öztürk, Fernando Louzada, Mario Pedrazzoli, Claudia R. Moreno

**Affiliations:** 1 Stress Research Institute, Stockholm University, 106 91 Stockholm, Sweden; 2 School of Arts, Science and Humanities, University of São Paulo, São Paulo 03828-000, SP, Brazil; 3 Human Chronobiology Laboratory, Department of Physiology, Federal University of Paraná, 81531-980 Curitiba, PR, Brazil; 4 Health, Life Cycles and Society Department, School of Public Health, University of São Paulo, 01246-904 São Paulo, SP, Brazil

**Keywords:** actigraph, circadian entrainment, high latitude, human, jetlag, light exposure, season, sleepiness, sleep schedule

## Abstract

Natural daylight exposures in arctic regions vary substantially across seasons. Negative consequences have been observed in self-reports of sleep and daytime functions during the winter but have rarely been studied in detail. The focus of the present study set out to investigate sleep seasonality among indoor workers using objective and subjective measures. Sleep seasonality among daytime office workers (*n* = 32) in Kiruna (Sweden, 67.86° N, 20.23° E) was studied by comparing the same group of workers in a winter and summer week, including work and days off at the weekend, using actigraphs (motion loggers) and subjective ratings of alertness and mood. Actigraph analyses showed delayed sleep onset of 39 min in winter compared to the corresponding summer week (*p* < 0.0001) and shorter weekly sleep duration by 12 min (*p* = 0.0154). A delay of mid-sleep was present in winter at workdays (25 min, *p* < 0.0001) and more strongly delayed during days off (46 min, *p* < 0.0001). Sleepiness levels were higher in winter compared to summer (*p* < 0.05). Increased morning light exposure was associated with earlier mid-sleep (*p* < 0.001), while increased evening light exposure was associated with delay (*p* < 0.01). This study confirms earlier work that suggests that lack of natural daylight delays the sleep/wake cycle in a group of indoor workers, despite having access to electric lighting. Photic stimuli resulted in a general advanced sleep/wake rhythm during summer and increased alertness levels.

## 1. Introduction

The arctic and polar regions present an interesting scenario for chronobiological investigation in which the photoperiod drastically changes throughout the year, having very long daylight hours during the summer but very short hours during the winter. This allows the effects of natural, extreme light/dark cycles on the synchronizing ability of biological timing systems to be explored. Although the biological timing system can be entrained through many biological pathways, light remains the main zeitgeber for the human species [1]. Exposure to daylight and solar timing in electrified in-door oriented societies show influences affecting human behavior and should therefore be recognized, although behaviors such as sleep timing is also regulated under social control [2]. When access to actigraphic sleep data could be obtained, data from the British Antarctic Survey base at Halley (2002–2003) confirmed the winter decrements in sleep quality and the winter delay in sleep timing, which were also observed in subjective diary data [3]. Interestingly, in one study condition, the research team presented a 1-h light pulse in the morning (4775 ± 1050 lux) at 08:30–09:30 in the winter [4]. The results for actigraphic sleep showed that in the bright light condition, sleep was shortened by 25 min, sleep onset was significantly advanced, and waking was advanced by 52 min. Improvements in ratings of sleep quality and morning alertness were also reported. Behavioral change in sleep timing across season was demonstrated by Kantermann and colleagues [5] using subjectively reported cross-sectional data, showing a delay of midsleep in winter at days off and a lengthening of sleep of about 20 min. Similar delays were demonstrated in objective sleep in connection to Daylight Saving Time using a longitudinal approach.

In polar areas midwinter insomnia increases [6], as demonstrated by reduced EEG derived measures of sleep efficiency, sleep length and slow wave sleep. In a large arctic population study in Tromsø (Northern Norway, 59° North), 17.6% of females and 9.0% of males reported insomnia during winter, but less other seasons [7]. A large questionnaire-based study in Tromsø reported only slight seasonal change in the sleep/wake cycle [8], although more sleep problems were evident in the winter [9]. Elevated sleepiness or fatigue in winter has been previously reported for arctic populations [10].

Sleep problems have been found to relate to a delayed circadian rhythm in winter [6], whereas sleep changes across seasons at a high latitude and lack of daylight is associated with more daytime tiredness [11]. Similar data is found among groups with scarce daylight exposure. Data suggest that underexposure to natural light at work may significantly impair sleep and wake disorders in low or non-light exposed workers [12,13]. In one study, sleep started 34 min later, and waking 35 min later, during the dark period (December) compared with brighter photoperiods [14].

The endogenous rhythms specific to each individual are masked during the productive days of the week and degree of entrainment enforced by social cues [15]. One such cue is the start and end of work, a daily fixed event. During the weekend, however, this cue is no longer present and the absence of a pre-arranged schedule causes biological rhythms to shift towards the endogenous phase, and the difference in sleep/activity rhythms between workdays and days off has also been referred to as social jetlag [16].

EEG recording is often unpractical and actimetry is widely used as a reliable method to study sleep in human populations [17,18,19]. Most studies in the field rely on parametrical analysis to summarize activity and sleep outcome measures, or when attempting to fit the observed rhythms with a cosinor function. Nevertheless, non-parametrical analysis does exist for chronobiology, such as the parameters proposed by Van Someren [20]: the 10 most active hours of the day (M10) contrasted with the 5 least active hours of the day (L5). These analyses have been used almost exclusively in a clinical context to determine sleep fragmentation of patients but could also be applied to healthy subjects exposed to different conditions, thereby providing a new perspective on sleep. The method provides an analysis of the timing of activity during the wake period, as well as identifying the timing of the 5-h lowest activity phase during sleep. Detailed data of seasonal change in sleep and light exposure of arctic workers is scarce, hitherto only briefly reported in questionnaires and diary-based studies of arctic populations describing more sleep delays and problems in winter. The main focus of the present study set out to investigate sleep seasonality among indoor workers using objective measures. An extreme real condition (arctic region) was chosen to see changes in sleep between seasons with different levels of natural light. Since sleep preferences are influenced by work hours [16], it was expected that days off would show greater seasonal differences.

In the present study, we report a bedtime delay in winter and bedtime advance in summer. We further suggest that seasonal sleep phase changes could be related to light exposure at different times of day.

## 2. Results

The studied population consisted of 32 day workers, comprising 15 males and 17 females, with a mean age of 45.6 ± 10.8 years, and the majority of which (28 workers) was married. While all workers had at least completed their high-school education, most had an improved formal education (65%). Workers took 16.2 ± 7.8 min to commute to work and their general self-evaluation of health was positive (80%). Six percent had kids aged 0–1.5 years, 19% had kids aged 1.5–7 years and 38% had kids living with them aged >7 years. In 50% of the cases, the parent reported spending less than 6 h per week in activities centered on kids (traveling to day care centers, reading, caring, supervision) and 87% of the parents spent less than 11 h.

**Figure 1:**
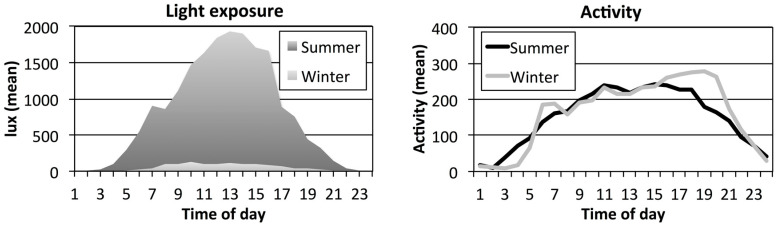
Mean hourly lux levels (**left**, units based on means of 1-min epochs of white light of arm worn light sensors) and mean hourly activity levels (**right**, units of 1-min epochs of arm worn accelerometer data) for day workers (*n* = 32) Sensor data in summer (dark grey/black) and in winter (light grey) across 24 h for seven days.

Mean light exposure levels peaked at 1846–1937 lux during midday (11:00–14:00 h) in summer, and levels exceeded 1000 lux between 08:00 to 15:00 h (in Figure 1 mean lux). In winter, light levels peaked at 109 lux (12:00–13:00 h) and remained at around 100 lux 07:00–14:00 h. Mean light levels at night during the sleep period reached 3–24 lux in summer (22:00–02:00 h) thereafter increased to reach above 500 lux after 05:00 h. In winter, light levels were below 10 lux from 21:00–05:00 h and then increasing to about 100 lux at 07:00 h.

**Figure 2:**
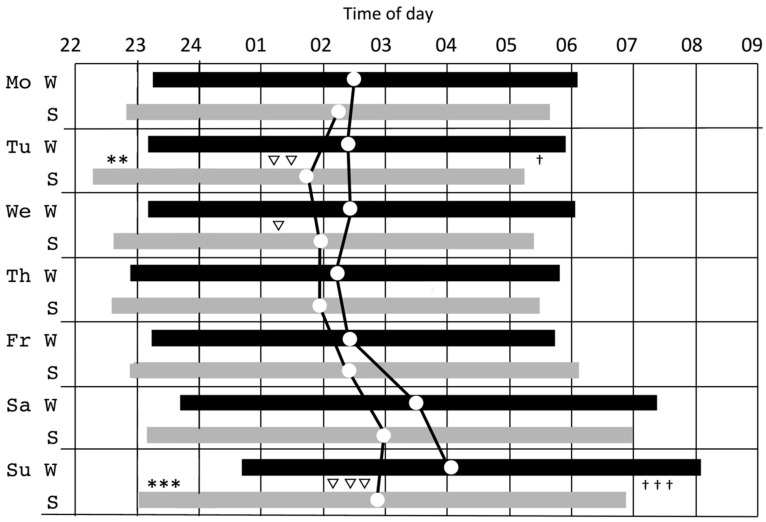
Mean sleep periods during the week (onset, offset, mid-sleep (circle dot)) during the week for winter (W, black bar) and summer (S, grey bar) with symbols marking seasonal differences between means for onset (*), mid-sleep (▽) and offset (†), (▽, † = *p* < 0.5; **, ▽▽ = *p* < 0.01; ***, ▽▽▽, ††† = *p* < 0.001).

Seasonal effects were found for sleep onset, for the factor day, and also for the season x day interaction (see Table 1). Across the full week (7 days), sleep onset was delayed 39 min in winter as compared to summer (see Figure 2). A difference between sleep onset for workdays (Sunday–Thursday) and days off (Friday–Saturday) can be noted. The seasonal difference for sleep onset amounted to a delay of 30 min on workdays in winter and 62 min on days off. The full week interaction effect and mean differences according to post hoc analyses indicate that sleep was positioned later in winter than in summer, especially on Saturdays and Mondays (see Figure 2).

**Figure 3:**
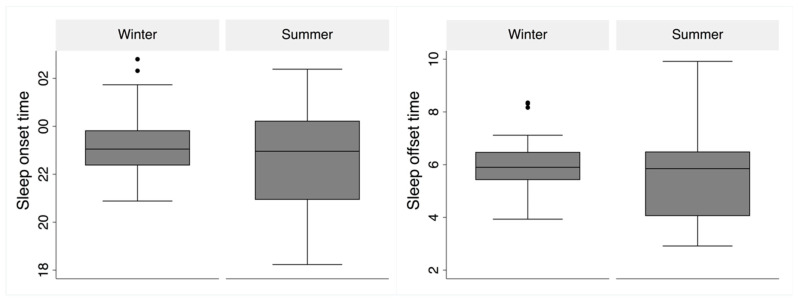
Box-plot of sleep onset (bottom left) and offset (bottom right) during workdays in winter and summer. The borders of the boxes represent the 25th (Q1) and 75th (Q3) quartiles, respectively. The boxes represent the interquartile range (IQR = Q3 - Q1). Whiskers are calculated as Q3 + (1.5 × IQR) and Q1 − (1.5 × IQR). Outliers outside that range are marked with dots in the figure.

A corresponding effect was found for sleep offset, which was delayed by 27 min in winter, showing differences between workdays and days off, with seasonal differences being greatest on Saturdays and Mondays. The mean seasonal difference in sleep offset on workdays showed a delay of 20 min and 41 min on days off in winter. The mean values and variation in sleep onset and offset for workdays are shown as boxplots in Figure 3 and suggest a seasonal difference and wider distribution in summer than in winter (Kolmogorov-Smirnov test for equality, *p* < 0.0001).

Mean sleep duration over 7 days was 6:25 ± 0.12 (hour:min) in winter and 6:37 ± 0.12 (hour:min) in summer, being 12 min longer in summer. Sleep duration was longer on days off than on workdays, with a similar pattern at both seasons. Sleep duration was not significantly longer on workdays in summer than in winter (6 min difference). The difference on days off (25 min) was not significant.

Mid-sleep was delayed by 32 min in winter and the seasonal difference was more apparent on days off (46 min) than on workdays (25 min). Calculating the mid-sleep difference between workdays and days off for both seasons revealed a social jetlag of 1.34 ± 0.14 h in winter and 0.86 ± 0.27 h in summer. Sleep efficiency calculated as the ratio between minutes awake and minutes asleep decreased in summer but remained around 92% for both seasons.

**Figure 4:**
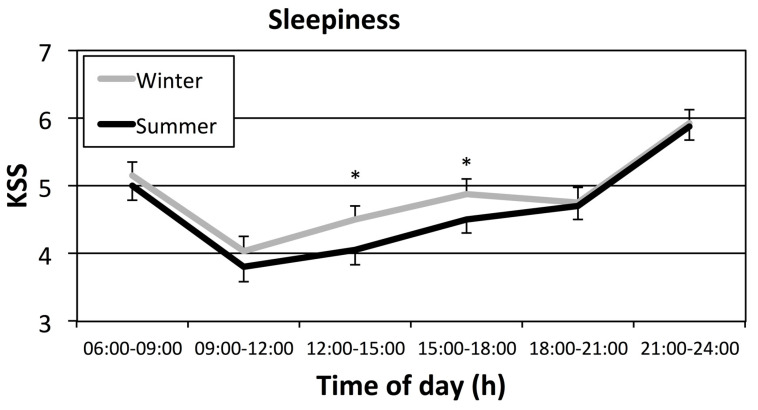
Median (+se) sleepiness (KSS—Karolinska Sleepiness Scale) during workdays in winter (grey line) and summer (black line) with asterisks marking seasonal differences between time periods (* = *p* < 0.05). Note that only part of the scale is used in figure (full scale 1–9).

Sleepiness (Figure 4) was more prominent in winter than in summer for two out of six evaluated time periods of the day: on workday afternoons (12:00–18:00 h).

No effects were observed for the onset of either the least active 5 h (L5) or most active 10 h (M10) between seasons (see Table 2). An effect of day was found for the onset of the period of L5 among days reflecting differences between workdays and days off. No differences were found for mean activity levels during M10 or during L5 between the summer and winter periods. Mean activity differed according to day.

Seasonal differences in light exposure were observed during the brightest 10 h (MB10) and the least bright 5 h (LB5). The illuminance was always higher during summer when compared to winter periods for both MB10 and LB5 (*p* < 0.0001, t-test; see Table 3). The onset of LB5 was about one hour later in summer.

**Figure 5:**
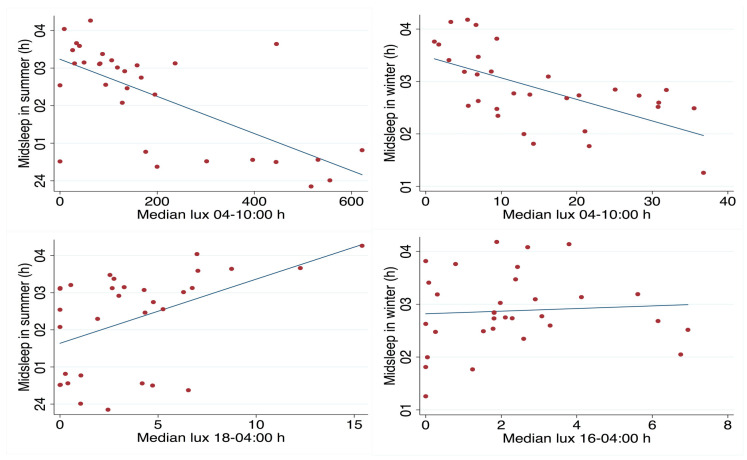
Scatter plot for day workers of mid-sleep hours (*y*-axis, time of day) against total light exposure (*x*-axis, median lux/h) during the morning hours (04–10:00 h) and fitted values in summer (**upper left**) and winter (**upper right**). Mid-sleep hours and light exposure during evening/night hours (18–04:00 h) in summer (**lower left**) and winter (**lower right**).

When comparing time of mid-sleep to median levels of light exposure throughout the day, three time windows were used to mark morning light exposure (04:00–09:59 h), midday exposure (10:00–17:59 h) and exposure in the evening and at night (18:00–03:59 h). Spearman’s rank correlation showed that morning light exposure negatively correlated with mid-sleep for both seasons (winter rho = −0.65, *p* < 0.001; summer rho = −0.58, *p* < 0.001; see Figure 5). Evening light exposure positively correlated with mid-sleep, but only in summer (rho = 0.47, *p* < 0.01; see Figure 5). The relationship between mid-sleep and midday exposure was non-significant for both seasons.

## 3. Discussion

Measurements in the arctic sample of studied day workers were taken at the most extreme photoperiods of natural light exposure, during midwinter darkness and bright midsummer. The difference in solar radiation strongly reflects light exposure of the workers, despite in-door work and the availability of electric lighting in the workplace, at home, in the form of street lighting, and in public areas. The present study indicates that, even in an industrialized population with a Western lifestyle, natural daylight exposure differences still have a marked influence on sleep timing and daytime sleepiness. It is likely that the dark season causes light deficits which increase the risk for circadian misalignment, and social jetlag, and may also contribute to other negative health consequences on sleep and mental health [13,21].

Results from the present study, based on measures of sleep, corroborate arctic studies which used questionnaires and diaries to define sleep timing [8,11]. In the diary study published by Friborg et al. [14], sleep started 34 min later, and wake-up 35 min later, during the dark period (December) compared with brighter photoperiods. In the present study, the seasonal difference reached similar levels of 39 min for sleep onset and 27 for offset, thus confirming a sleep delay in an arctic region with objective sleep measures. Friborg et al. used a sample of students likely to have some degree of freedom, akin to the day workers in Kiruna, in choosing sleeping times because they worked flexi-time. However, the former study found a decrease in sleep efficiency, not confirmed in the present study. In the present study, seasonal sleep efficiency differences seem small but decreased efficiency was observed in summer. Stricter, more firmly set work hours might explain why some studies have only found slight arctic seasonal changes in sleep [8]. Our data complements population questionnaire data by demonstrating seasonal time of day effects in non-closed communities. From our data, we may conclude that elevated sleepiness in winter affects early and late afternoon, periods that coincide with time spent at work. This increased sleepiness is probably related to the multiple influences of social jetlag, lack of alerting daylight, sleep deficit, depressed mood and seasonal affective disorder often reported at high latitudes in winter [22].

These results might be explained by the scenario where an early sleep phase in summer could be related to the strong circadian stimulus influence of the morning natural light exposure at the advancing phase of the day observed in office work [21]. Even the rather short trip to work could provide a strong light pulse. This influence is further supported by the stronger awaking signal in the bedroom by light during sleep in summer, as demonstrated by the difference in LB5 light levels (see Table 3). This scenario is supported by indications of the high negative correlation between midsleep and light exposure during morning hours, although it is important to recognize that sleep behavior also affects light measures. Borisenkov [23] found that an early sunrise during the period of January to May at an arctic latitude was the best predictor for changes towards early chronotype characteristics. Earlier findings have stressed the importance of the first photic stimuli after darkness. For example, Crowley has, in experimental settings, shown that 30 min of artificial bright light during early morning promoted 75% of a phase advance shift as compared to 2 h bright light exposure later [24]. Similarly, also when calculating a more precise prediction of the circadian stimulus for the human eye comparing summer and winter morning light exposures of office workers, daylighting is found to influence sleep quality as well as circadian phasor magnitude measures based on activity measures [21]. Nevertheless, the differences between winter and summer were not observed in the least active 5 h (L5) and the most 10 active hours (M10) of the day. In other words, although the workers slept later in the winter, they showed a similar activity patterns according to the season. We only found a day effect, which could be related to the workdays versus days off.

An additional influence may be the stabilizing effect of light exposure during the middle of the day in summer. In summer, the influence of photic history from daytime bright light exposure weakens the evening suppression of melatonin despite light exposure in the evening [25,26]. Furthermore, evening suppression of melatonin in summer might truncate the melatonin-producing period at night, but not necessarily cause a delay shift in the circadian phase [27], a phenomenon which might have occurred in our day workers. The lack of sleep delay observed in summer resembles findings from studies comparing groups subjected to different light exposure levels. Wright and colleagues [28] compared students in a modern urban indoor environment with individuals at a non-electrified outdoor campsite in Colorado, observing advanced sleep onset outdoors.

From a chronobiological and light perspective, other scenarios have been discussed to explain seasonal differences. The sleep/wake cycle may be more delayed at high latitudes during summer than winter due to strong natural light exposure during the phase-delaying part of the Phase Response Curve [29,30]. In theory this would be the time of day when the core body temperature shows a decline. Such exposure would further suppress evening melatonin, which could in turn suppress sleepiness and thus delay bedtime, as well as having direct alerting effects. This was indeed observed by Paul et al., studying a group of indoor-working military personnel at a station 58° North in Alaska [31]. The delay of sleep timing in summer was attributed to evening light exposure. Nevertheless, our study showed the opposite, possibly owing to the differences in timing of natural light. A lack of morning light exposure in the military personnel in Paul’s study, with no travel time to work and indoor work, might have influenced sleep timing (and quality) differently. Moreover, in the present study, all workers were exposed to nighttime light at home.

A phase-delayed sleep cycle in winter occurs when the photic zeitgebers are weak, resulting in a lack of entrainment and stronger impact from an endogenous delaying (free running) rhythm [32]. However, we observed no increase in the standard deviation for sleep onset in winter, which might be expected if the endogenous impact were significant. The delayed sleep and narrow sleep distributions for sleep timing in winter observed in the present study are likely due to the combined effects of a lack of morning light pulses, photic history impact, and the presence of artificial lighting in the evening. Summer sleep timing would thus be secondary to phase-advancing morning photic stimuli and continued midday photic pulses, forming a photic history that reduces the impact of the combined evening artificial and natural light exposure. Even if mean levels produce small delays in sleep timing, constant natural light around the clock would increase individual differences in light exposure and influence sleep timing, as observed in the present study (see Figure 3).

The delayed sleep onset was significantly more pronounced on Saturday and Monday nights in winter, as shown in Figure 2. It could be argued that the common delay of sleep at weekends and possible delay in rhythm are more difficult to re-entrain in winter. Mid-sleep data suggests readjustment does not occur until mid-week in winter as compared to the summer. Diary data from the same population indicate that daytime sleepiness is elevated among groups with a later sleep phase, who risk shorter and less refreshing sleep [33]. In summary, the winter period, as compared to summer, would likely elevate the risk of inducing sleep depth and daytime sleepiness during the workweek.

Limitations of this study include the fact that circadian markers were not measured to determine whether sleep/wake outcome measurements corresponded to circadian rhythms. Due to the small sample size, it was not meaningful to further investigate individual differences or reactions by particular diurnal types. The lack of precision of actigraphic measures precluded detailed study of seasonal sleep latency differences and insomnia within this healthy population. Also, it cannot be ruled out that seasonal changes in sleep reported here might not only be influenced by natural light conditions but also clock time changes in connection to Daylight Saving Time (DST). Even though the effects from DST should have declined by late December and late June, reports have indicated that extreme chronotypes could have difficulties adjusting to a new clock time [5].

In this study, we suggest that a lack of strong natural daylight in the morning promotes a delay in the sleep/wake rhythm among daytime workers. In summer, where daylight exposure is extended, the sleep/wake rhythm generally becomes less delayed than in winter, but more varied photic stimuli patterns may increase individual differences.

## 4. Study Population and Methods

Arctic day- and shift-workers at an iron ore mine in the arctic region of Kiruna, Sweden (67.86° N, 20.23° E) were initially asked by e-mail to fill out online questionnaires for both winter and summer, reporting seasonal differences in sleep and mood. A group of 574 day workers responded, giving background information on demographics, sleep, work, and health. The present study included a subgroup of 32 day workers that had signed up in response to a second e-mail to be part of an actigraph and diary study. The study group worked in-doors Monday through Friday with two days off during weekends. Workers had a flexi-time 8 h work schedule, allowing them to start and end work according to personal preference. A lunch break commonly extended the workday to around 9 h a day. About one third of the workers were engineers and geologists, another third managers and coordinators, while the remainder worked in human relations, development and control tasks.

Workers were informed, either in groups or individually, about the measuring procedure of the data collection, which included instructions on actigraphy and diary use. Data were collected in June 2014 and again in December 2014. The winter measurements were obtained during polar nights in December, when the sun does not rise above the horizon. In the outdoor environment during winter, natural daylight never yields more than 1 kWh/m^2^ in global energy from solar radiation. Summer measurements were obtained during polar days when the sun never dips below the horizon. 

The workers reported no treatment for sleep problems or use of medication that could interfere with sleep. Data collection using diaries and actigraphs was performed according to a procedure starting on a Wednesday and finishing one week later, yielding seven consecutive nights of records, including two days off. The study was approved by the Regional Ethics Review Board, Stockholm, Sweden (protocol n° 2012/2145-31/3) in accordance with the ethical standards laid down in the 1964 Declaration of Helsinki and its later amendments [34]. All procedures were carried out with an adequate understanding, and all workers provided written informed consent.

The online questionnaire included questions on demographics (sex and age), work (commuting time, work schedule satisfaction) and health (sleep habits, self-reported health, and medication).

Actigraphs (motion loggers) were used to register participants’ activity, as well as light exposure in lux. The actigraph (MotionWatch 8, CamNtech Ltd., Cambridge, UK) was worn on the non-dominant wrist. The watch incorporated an expandable wristband allowing use over outdoor clothing. Activity-rest data were exported to the Philips Respironics software (Respironics Actiware, Bend, OR, USA, version 5.71.0^®^) for sleep scoring. A medium sensitivity was used for sleep analyses and scoring was adjusted using sleep diary records of bedtimes and rise times. Analyses identified sleep onset (10 undisturbed epochs), sleep offset, sleep duration, wake duration after sleep onset (WASO), and sleep efficiency (ratio between sleep duration and time in bed). Mid-sleep time was derived by adding half of the time difference between actigraphic sleep onset time and time of final awakening to time of sleep onset. Each subject contributed 14 days, but 6.9% of the sleep periods were considered missing or incomplete. No subject had more than two days missing. Raw data was collapsed from 1-min bins to 1-h bins to produce smoother diurnal profiles, using mean levels of light (logged) and mean activity. In this analysis, one subject was withdrawn due to a malfunctioning light meter. The raw data was later converted to text file format (.txt) and subsequently imported into Matlab (R2016b), which was used to perform further analytical and statistical comparisons.

Sleepiness during seven days was reported using the Karolinska Sleepiness Scale (KSS; [35,36]). KSS is a Likert-like scale assumed to be an interval scale with equal-sized intervals between each score. This scale ranges from 1 (very alert) to 9 (very sleepy, fighting sleep, an effort to keep awake). In the present study, workers provided KSS average ratings for six intervals during each day of study (time intervals: 06:00–09:00, 09:00–12:00, 12:00–15:00, 15:00–18:00, 18:00–21:00 and 21:00–23:00 h). Workers were instructed to fill in the diary twice a day. In the analysis, medians were used for workdays, and seasonal differences between seasons were calculated for single time intervals by use of Wilcoxon signed-rank test.

A non-parametrical analysis of circadian rhythm was used to calculate the 10 most active hours (M10) and the 5 least active hours of the day (L5). For each day, a sliding window of 10 or 5 h, respectively, was run across the data points, giving an average activity within this timeframe indicating the most active or inactive period. In order to account for the missing data in the dataset (minutes workers did not wear the actigraph), a threshold was set to include a maximum of 30% of missing data. If a given window had more blank data, it was discarded and the window moved along. The same process was performed for light measurements. We also compared the mean exposure and the time onset of the 10 brightest light hours (MB10) and the 5 least bright light hours (LB5) during winter and summer.

Statistical analyses were performed using Stata14 (Copyright 1985–2015 StataCorp LP©, College Station, TX, USA). A mixed model repeated measures design including the factors season and day (or workdays vs. days off) was used in tables, yielding a Chi-square output statistic for contrasts of marginal linear predictions. The mixed model repeated approach yielding Chi2 values was chosen since it allowed missing observations within a subject. Chi2-values were later transformed to represent F-values (Chi2/df). If a significant main effect was observed, pairwise comparisons of adjusted predictions were used to establish differences between means for single days or time slots in different seasons or by use of t-test. The Spearman’s rank correlation coefficient was calculated when comparing individual means for mid-sleep and median light exposure across three periods for workdays during the morning (04–09:59 h), midday (10–17:59 h), and evening/night (18–03:59 h).

## Figures and Tables

**Table 1:** Sleep data including means (m) and standard error (se) from actigraphy among day workers (*n* = 32) for one full week and separated for workdays (5 days, Monday–Friday) and days off (2 days, Saturday–Sunday). A mixed model repeated measures design tested the effect of factor Season (winter vs. summer), Day (7 days—full week, 5 days—workdays and 2 days—days off) and interaction Season and Day yielding F–values (*F*) and level of significance (*p*). Significant *p*–values in bold.

	Winter		Summer		Season		Day		Season and Day	
	m	se	m	se	*F*	*p*	*F*	*p*	*F*	*p*
Sleep onset (hh:min)										
Full week	23:26	0.20	22:47	0.20	17.9	**0.0001**	7.5	**0.0001**	2.7	**0.0133**
Workdays	23:05	0.21	23.35	0.21	9.4	**0.0001**	1.3	0.0998	1.0	0.1998
Days off	24:06	0.24	23:04	0.25	9.5	**0.0001**	0.74	**0.0426**	0.67	**0.0444**
Sleep offset (hh:min)										
Full week	06:25	0.22	05:58	0.22	2.9	**0.0154**	16.1	**0.0001**	1.5	0.4682
Workdays	05:53	0.18	05:32	0.18	4.6	**0.0024**	1.2	0.1325	1.8	**0.0328**
Days off	07:40	0.24	06:59	0.26	2.9	**0.0162**	0.25	0.2200	0.24	0.2310
Sleep duration (h)										
Full week	6.42	0.22	6.62	0.22	2.0	**0.0437**	7.0	**0.0001**	1.5	0.1890
Workdays	6.28	0.12	6.38	0.12	0.6	0.2858	0.21	0.8651	1.1	0.1510
Days off	6.81	0.20	7.22	0.21	5.0	0.0848	0.28	0.1985	0.31	0.2991
Mid-sleep (hh:min)										
Full week	02:52	0.10	02:20	0.10	15.0	**0.0001**	18.6	**0.0001**	2.4	**0.0232**
Workdays	02:29	0.18	02:04	0.18	8.2	**0.0001**	1.5	0.0549	1.4	0.0698
Days off	03:47	0.20	03:01	0.21	7.8	**0.0001**	0.46	0.0986	0.42	0.1102
Sleep efficiency (%)										
Full week	90.0	0.55	90.7	0.55	2.5	**0.0256**	1.8	0.1008	1.9	0.0794
Workdays	90.2	0.57	90.9	0.57	2.2	**0.0350**	0.60	0.4661	0.71	0.3702
Days off	89.6	0.69	90.0	0.72	0.13	0.6112	0.01	0.8180	0.04	0.6122

**Table 2:** Activity data (means ± standard error) for the least active 5 h (L5) and the most active 10 h (M10) of the day. A mixed model repeated measures design tested the effect of factor Season (winter vs. summer), Day (7 days-full week), and interaction Season and Day yielding F-values (*F*) and level of significance (*p*). Significant *p*–values in bold.

	Winter		Summer		Season		Day		Season and Day	
	m	se	m	se	*F*	*p*	*F*	*p*	*F*	*p*
Onset L5 (hh:min)	24:18	0.18	24:03	0.18	1.0	0.1483	13.9	**0.0001**	2.1	0.0543
Onset M10 (hh:min)	08:45	0.31	08.05	0.23	0.56	0.2877	1.6	0.1514	0.32	0.9297
Mean activity M10	256	10	246	10	0.50	0.3167	7.0	**0.0001**	0.58	0.7479
Mean activity L5	7.82	0.99	8.21	1.01	0.09	0.6713	0.39	0.8857	1.2	2867

**Table 3:** Light data, mean (m) and standard error (se) for the least bright 5 h of exposure (LB5) and the brightest 10 h of exposure (MB10) of the day in winter and summer. Seasonal differences (Season) calculated by t-test (*t*-value) and level of significance (*p*). Significant *p*–values in bold.

	Winter			Summer		Season	
	m	se	m		se	*t*-value	*p*
Mean MB10 Light (lux)	95.9	45	1673		1123	6.01	**0.0001**
Mean LB5 Light (lux)	0.37	0.13	3.67		0.61	5.11	**0.0001**
Onset MB10 Light (hh:min)	08:02	1.44	07:48		1.72	0.51	0.6134
Onset LB5 Light (hh:min)	24:11	1.44	23:10		1.72	2.67	**0.0162**
